# On the exact continuous mapping of fermions

**DOI:** 10.1038/s41598-018-31162-6

**Published:** 2018-08-28

**Authors:** Andrés Montoya-Castillo, Thomas E. Markland

**Affiliations:** Department of Chemistry, Stanford University, Stanford, California 94305 USA

## Abstract

We derive a rigorous, quantum mechanical map of fermionic creation and annihilation operators to continuous Cartesian variables that exactly reproduces the matrix structure of the many-fermion problem. We show how our scheme can be used to map a general many-fermion Hamiltonian and then consider two specific models that encode the fundamental physics of many fermionic systems, the Anderson impurity and Hubbard models. We use these models to demonstrate how efficient mappings of these Hamiltonians can be constructed using a judicious choice of index ordering of the fermions. This development provides an alternative exact route to calculate the static and dynamical properties of fermionic systems and sets the stage to exploit the quantum-classical and semiclassical hierarchies to systematically derive methods offering a range of accuracies, thus enabling the study of problems where the fermionic degrees of freedom are coupled to complex anharmonic nuclear motion and spins which lie beyond the reach of most currently available methods.

## Introduction

The Meyer-Miller-Stock-Thoss (MMST) approach provides an exact prescription to map a Hamiltonian consisting of discrete states to one in terms of continuous Cartesian phase space variables (positions and momenta). Originally introduced as the “classical electron” model^1^, this approach was later generalized and shown to be a rigorous quantum mechanical representation^2^. The ability to represent the Hamiltonian in terms of continuous Cartesian phase space variables facilitates the use of classical-like trajectories to obtain quantum mechanical information via exact path integral approaches as well as quantum-classical and semiclassical approximations. In particular, the MMST mapping has provided the cornerstone for the development and application of a large family of nonadiabatic methods with a range of accuracies based on quantum-classical^3–9^ and semiclassical^10–26^ approximations to the mapped propagator. However, while the MMST protocol provides a route to map Hamiltonians containing discrete states, for those that include fermionic creation and annihilation operators an exact Cartesian mapping has remained elusive. The lack of a mapping approach for fermionic operators has thus prevented the application of similar approaches to many-fermion problems where the discrete energy levels are so numerous as to create continua, as is the case for processes near metallic and semiconducting interfaces.

In principle, one could apply the MMST approach to problems containing fermionic creation and annihilation operators by expressing the second-quantized operators in terms of outer products of the many-body basis. However, the Hilbert space constructed using *M* single-particle orbitals contains 2^*M*^ many-body states, which, upon mapping, results in twice this number of phase space variables (2^*M*+1^). This exponential scaling with the number of single-particle orbitals is mildly ameliorated in cases involving a fixed number of fermions, *N*, where the 2^*M*^ dimensional Hilbert space can be limited to the *M*!/*N*!(*M*−*N*)! dimensional Fock space, but in practice this still renders the MMST treatment infeasible in most cases.

To obviate this highly unfavorable scaling with the number of single-particle orbitals, one could instead consider directly mapping the fermionic creation and annihilation operators, which naturally encode the antisymmetry of the fermionic wavefunction by virtue of their anticommutivity. Since this would allow linear scaling in the number of orbitals, a number of continuous mappings have been suggested based either on fermion coherent states^27–33^ or physically motivated connections in the classical limit^34–37^ (for example, by noting the similarity between fermionic anticommutivity and vector cross products).

Here, we derive a rigorous mapping that provides a general approach to represent fermionic creation and annihilation operators as continuous Cartesian phase space variables. Our map thus provides an exact starting point for the application of the entire arsenal of quantum-classical and semiclassical techniques to investigate the statics and dynamics of problems involving many fermions. These methods can be used to elucidate physical processes in systems ranging from electrochemical interfaces to nanojunctions and strongly correlated materials.

## Fermion mapping

Our objective is to map a general many-fermion Hamiltonian of the form

1 $$\hat{H}=\sum _{j,k}\,{h}_{j,k}\,({\boldsymbol{\Gamma }}){\hat{c}}_{j}^{\dagger }{\hat{c}}_{k}+\frac{1}{2}\sum _{j,k,l,m}\,{U}_{jk,lm}\,({\boldsymbol{\Gamma }})\,{\hat{c}}_{j}^{\dagger }{\hat{c}}_{k}^{\dagger }{\hat{c}}_{m}{\hat{c}}_{l},$$

where ${\hat{c}}_{j}^{\dagger }$ and *ĉ*_*j*_ are the fermionic creation and annihilation operators for the *j*^th^ single-particle orbital, *h*_*j,k*_(**Γ**) are the matrix elements of the single-particle part of the Hamiltonian and *U*_*jk*,*lm*_(**Γ**) are those of the two-body component. In the general case, these matrix elements could themselves depend on an additional set of continuous or discrete degrees of freedom, **Γ** ≡ {**P**, **Q**, |*r*〉 〈*s*|}. This type of Hamiltonian encompasses a broad class of systems, including those where the fermionic dynamics are coupled to complex atomistic or spin degrees of freedom. For example, Eq. (1) encompasses the Hubbard^38–40^, Anderson impurity^41^, and Holstein^42^ models, which form the basis of our description of processes such as superconductivity in correlated materials^43–46^, charge conduction in nanoscopic junctions^47–49^, and polaron formation^50,51^.

To introduce an exact continuous mapping of Eq. (1), we first express the fermionic operators in terms of two level system (spin 1/2) operators. While spins can describe the occupied and unoccupied states of a single-particle orbital, spins on different sites do not naturally anticommute with each other. Hence, it is necessary to encode the fermionic anticommutivity, which can be done formally by employing the Jordan-Wigner (JW) transformation^52,53^. This transformation has previously been used to enable the solution of fundamental problems in magnetism^53^ and, more recently, the quantum simulation of fermionic Hamiltonians^54–58^. By using the JW transformation, one can exactly map a set of *M* second quantized fermionic operators corresponding to the creation or annihilation of a fermion in a single-particle orbital to *M* spins arranged in a one-dimensional (1D) lattice,

2a $${\hat{c}}_{j}\mapsto \hat{F}\mathrm{(0},j){\sigma }_{j}^{-},$$

2b $${\hat{c}}_{j}^{\dagger }\mapsto \hat{F}\mathrm{(0},j){\sigma }_{j}^{+},$$

where

3 $$\hat{F}(j,k)\equiv \prod _{l=\,{\rm{\min }}\,[j+1,k+\mathrm{1]}}^{{\rm{\max }}\,[j-1,k-\mathrm{1]}}\,f({\sigma }_{l}^{z})$$

is the nonlocal operator that imposes the fermionic anticommutivity, *j*, *k* ∈ {1, …, *M*}, and $\hat{F}(j,j)=\hat{F}(j,j+\mathrm{1)}=1$. The operators for the *j*^*th*^ spin are

4a $${\sigma }_{j}^{+}=|{1}_{j}\rangle \langle {0}_{j}|,$$

4b $${\sigma }_{j}^{-}=|{0}_{j}\rangle \langle {1}_{j}|,$$

4c $${\sigma }_{j}^{z}=|{1}_{j}\rangle \langle {1}_{j}|-|{0}_{j}\rangle \langle {0}_{j}|.$$

It is often convenient to set $f({\sigma }_{l}^{z})=-\,{\sigma }_{l}^{z}$^54^, which trivially yields the correct behavior when the problem is treated quantum mechanically. However, the function $f({\sigma }_{l}^{z})$ in Eq. (3) can be shown to give the exact quantum mechanical solution as long as it outputs −1 when the *l*^*th*^ single-particle orbital is occupied and +1 otherwise. This is important to note since a different functional form may prove more advantageous if one were to treat the mapped many-fermion Hamiltonian using quantum-classical or semiclassical theories, where ${\sigma }_{l}^{z}$ could take values different from ±1^59^. However, as long as $f({\sigma }_{l}^{z})$ satisfies the above requirement, it is simple to confirm that the JW transformation exactly reproduces the fermionic anticommutation relations, $\{{\hat{c}}_{j},{\hat{c}}_{k}^{\dagger }\}=\{\hat{F}\mathrm{(0,}\,j){\sigma }_{j}^{-},\hat{F}\mathrm{(0,}\,k){\sigma }_{k}^{+}\}={\delta }_{j,k}$^53^.

It is insightful to briefly consider how the JW transformation encodes the fermionic anticommutivity via the ordering of spins along a 1D chain. By arranging the fermionic single-particle orbitals used to construct the many-body Hilbert space along a 1D chain, the JW transformation keeps a record of the normal ordering of the many-body basis. By additionally including the operator, $\hat{F}(j,k)$, which exploits the normal ordering of the many body-basis mirrored in the 1D chain arrangement, the JW transformation imposes anticommutivity on the spins, which otherwise commute. This allows the mapped operators to act on the many-body basis in the same way as the original fermionic creation and annihilation operators.

We now express the discrete spin states resulting from the JW transformation in terms of bosonic degrees of freedom. To achieve this, we employ the Schwinger theory of angular momentum^60^ to express spin operators as coupled bosons. This map was initially developed to easily obtain rotation matrices and Clebsch-Gordan coefficients^60^ and subsequently exploited in semiclassical theories of magnetism^61,62^. The Schwinger transformation maps a single spin labelled by index *j* to two coupled bosons, to which we refer as the *α* and *β* modes,

5a $${\sigma }_{j}^{+}\, \mbox{"} \mapsto  \mbox{"} \,{\hat{b}}_{j\beta }^{\dagger }{\hat{b}}_{j\alpha },$$

5b $${\sigma }_{j}^{-}\, \mbox{"} \mapsto  \mbox{"} \,{\hat{b}}_{j\alpha }^{\dagger }{\hat{b}}_{j\beta },$$

5c $${\sigma }_{j}^{z}\, \mbox{"} \mapsto  \mbox{"} \,{\hat{b}}_{j\beta }^{\dagger }{\hat{b}}_{j\beta }-{\hat{b}}_{j\alpha }^{\dagger }{\hat{b}}_{j\alpha }.$$

Here the quotation marks indicate, as shown in Supplementary Material, Sec. 1, that the Schwinger transformation is not exact on an operator level. However, as shown in the Supplementary Material, Sec. 1, the map becomes exact when one restricts it to the physical basis, i.e., the joint single-excitation subspace consisting of the states for which the *α* mode is in its first excited state and the *β* mode is in its ground state, and vice versa, for every *j*. Hence, while the Schwinger theory of angular momentum does not constitute an isomorphism on the operator level, it is an *exact* isomorphism on the matrix element level when they are evaluated using the basis consisting of only the single-excitation manifold for each *j*. Indeed, this can be confirmed by using the mapped spin ladder operators, ${\sigma }_{j}^{+}$ and ${\sigma }_{j}^{-}$, to construct the spin polarization and unit operators, ${\sigma }_{j}^{z}={\sigma }_{j}^{-}{\sigma }_{j}^{+}-{\sigma }_{j}^{+}{\sigma }_{j}^{-}$ and ${{\bf{1}}}_{j}={\sigma }_{j}^{-}{\sigma }_{j}^{+}+{\sigma }_{j}^{+}{\sigma }_{j}^{-}$, and noting that the correct form of the latter can be recovered when excitations outside the single-excitation manifold are eliminated. We are now in a position to combine the Schwinger map with the JW transformation to yield an exact bosonic representation of fermionic matrix elements.

As we demonstrate in the Supplementary Material, Sec. 2, using Eqs. (2), (3) and (5), one can derive an exact isomorphic representation of fermionic matrix elements in terms of bosonic ones,

6 $$\langle \tilde{{\bf{n}}}|\hat{O}(\{{\hat{c}}_{j}^{\dagger },{\hat{c}}_{j}\})|\tilde{{\bf{n}}}^{\prime} \rangle \mapsto \langle {\bf{n}}|\hat{O}(\{{\hat{b}}_{j\gamma }^{\dagger },{\hat{b}}_{j\gamma }\})|{\bf{n}}^{\prime} \rangle .$$

Here, the ordered fermionic occupation number basis, $\tilde{{\bf{n}}}\equiv \{{\tilde{n}}_{1},{\tilde{n}}_{2},\mathrm{...},{\tilde{n}}_{M}\}$ where ${\tilde{n}}_{j}\in \mathrm{\{0,}\,\mathrm{1\}}$, is mapped to its bosonic counterpart, ${\bf{n}}\equiv \{{n}_{1\alpha },{n}_{1\beta },{n}_{2\alpha },{n}_{2\beta },...,{n}_{M\alpha },{n}_{M\beta }\}$, where ${n}_{j\beta }={\mathop{n}\limits^{ \sim }}_{j}$ and ${n}_{j\alpha }=1-{\mathop{n}\limits^{ \sim }}_{j}$. An arbitrary fermionic operator $\hat{O}(\{{\hat{c}}_{j}^{\dagger },{\hat{c}}_{j}\})$ can then be written in terms of bosonic operators $\hat{O}(\{{\hat{b}}_{j\gamma }^{\dagger },{\hat{b}}_{j\gamma }\})$, where $\gamma \in \{\alpha ,\beta \}$, using,

7a $${\hat{c}}_{j}\, \mbox{"} \mapsto  \mbox{"} \,\hat{F}(0,j){\hat{b}}_{j\alpha }^{\dagger }{\hat{b}}_{j\beta },$$

7b $${\hat{c}}_{j}^{\dagger }\, \mbox{"} \mapsto  \mbox{"} \,\hat{F}(0,j){\hat{b}}_{j\beta }^{\dagger }{\hat{b}}_{j\alpha },$$

where

8 $$\hat{F}(j,k)\, \mbox{"} \mapsto  \mbox{"} \,\prod _{l=\,min\,[j+1,k+1]}^{max\,[j-1,k-1]}\,f({\hat{b}}_{j\beta }^{\dagger }{\hat{b}}_{j\beta }-{\hat{b}}_{j\alpha }^{\dagger }{\hat{b}}_{j\alpha }).$$

Here the quotation marks around the map symbol emphasize that the transformation in Eqs. (7) and (8) only works on the operator level if one eliminates all the excitations that lie outside of the physical subspace of the *α* and *β* modes, which one can exactly enforce by using the physical basis (Supplementary Material, Sec. 2). Equations (6)–(8) thus provide a formal prescription to exactly obtain the matrix elements of fermionic operators from an isomorphic bosonic representation.

Finally, it is worth noting that, while not our primary focus, fermion-to-boson maps are themselves of interest for both practical and fundamental reasons^63,64^. The current map achieves this in a simple form that exactly recovers the correct matrix structure of the many-fermion problem and avoids the issues in some previous ones that result in infinite expansions of fermion operators in terms of bosonic ones^63,64^. We also note that other spin-to-boson maps are possible, such as the Holstein-Primakoff^65^ and Matsubara-Matsuda^66^ transformations. In the Supplementary Material, Sec. 3, we derive the Cartesian maps of fermionic operators that would be obtained using these transformations. We show that the former can also be used to obtain a phase space map that exactly recovers the matrix structure of the many-fermion problem, albeit at the price of cumbersome nonlinearities in the form of square roots of occupation number operators. For the latter, while we provide the Cartesian map that could be generated from it, we also show that this map is unable to yield an exact Cartesian representation of fermionic operators or their matrix elements. However, we suggest how it could be used in a controlled manner in a path integral treatment of many-fermion problems.

We are now in a position to map the fermionic operators $\{{\hat{c}}_{j}^{\dagger },{\hat{c}}_{j}\}$ to Cartesian phase space variables $\{{\hat{q}}_{j\gamma },{\hat{p}}_{j\gamma }\}$. We achieve this by expressing the bosonic operators $\{{\hat{b}}_{j\gamma }^{\dagger },{\hat{b}}_{j\gamma }\}$ in Eqs. (7) and (8) in their phase space representation,

9a $$\hat{b}=(\hat{q}+i\hat{p})/\sqrt{2},$$

9b $${\hat{b}}^{\dagger }=(\hat{q}-i\hat{p})/\sqrt{2}.$$

This yields,

10a $${\hat{c}}_{j}\, \mbox{"} \mapsto  \mbox{"} \,\frac{1}{2}\hat{F}(0,\,j)({\hat{q}}_{j\alpha }-i{\hat{p}}_{j\alpha })({\hat{q}}_{j\beta }+i{\hat{p}}_{j\beta }),$$

10b $${\hat{c}}_{j}^{\dagger }\, \mbox{"} \mapsto  \mbox{"} \,\frac{1}{2}\hat{F}(0,\,j)({\hat{q}}_{j\beta }-i{\hat{p}}_{j\beta })({\hat{q}}_{j\alpha }+i{\hat{p}}_{j\alpha }),$$

where

11 $$\hat{F}(j,k)\, \mbox{"} \mapsto  \mbox{"} \,\prod _{l=\,min\,[j+1,k+1]}^{max\,[j-1,k-1]}\,f({\hat{m}}_{l\beta }-{\hat{m}}_{l\alpha }),$$

and

12 $${\hat{m}}_{j\gamma }=\frac{1}{2}({\hat{q}}_{j\gamma }^{2}+{\hat{p}}_{j\gamma }^{2}-1)$$

can be recognized as the occupation number operator corresponding to the $\gamma \in \{\alpha ,\beta \}$ boson labelled by index *j*. Equations (10) and (11) then allow us to map $\hat{H}$ in Eq. (1) to a Cartesian representation, $\hat{H}(\{{\hat{c}}_{j}^{\dagger },{\hat{c}}_{j}\})\mapsto \hat{H}(\{{\hat{q}}_{j\gamma },{\hat{p}}_{j\gamma }\})$, expressed in terms of the continuous coordinates and momenta of fictitious particles,

13 $$\begin{array}{ccc}\hat{H} & = & \frac{1}{2}\sum _{j,k}\,{h}_{j,k}({\boldsymbol{\Gamma }})\hat{F}(j,k)({\hat{q}}_{j\beta }-i{\hat{p}}_{j\beta })({\hat{q}}_{j\alpha }+i{\hat{p}}_{j\alpha })({\hat{q}}_{k\beta }+i{\hat{p}}_{k\beta })({\hat{q}}_{k\alpha }-i{\hat{p}}_{k\alpha })\\  &  & +\,\frac{1}{{2}^{3}}\sum _{j,k,l,m}{U}_{jk,lm}({\boldsymbol{\Gamma }}){\rm{s}}{\rm{g}}{\rm{n}}(k-j){\rm{s}}{\rm{g}}{\rm{n}}(m-l)\hat{F}(j,k,l,m)({\hat{q}}_{j\beta }-i{\hat{p}}_{j\beta })({\hat{q}}_{j\alpha }+i{\hat{p}}_{j\alpha })\\  &  & \times \,({\hat{q}}_{k\beta }-i{\hat{p}}_{k\beta })({\hat{q}}_{k\alpha }+i{\hat{p}}_{k\alpha })({\hat{q}}_{m\beta }+i{\hat{p}}_{m\beta })({\hat{q}}_{m\alpha }-i{\hat{p}}_{m\alpha })({\hat{q}}_{l\beta }+i{\hat{p}}_{l\beta })({\hat{q}}_{l\alpha }-i{\hat{p}}_{l\alpha }),\end{array}$$

where

14 $$\hat{F}({\bf{r}})\equiv \hat{F}({s}_{1},{s}_{2})\hat{F}({s}_{3},{s}_{4})\times \mathrm{...}\times \hat{F}({s}_{2N-1},{s}_{2N})$$

is the many-index generalization of the antisymmetry operator, ${\bf{s}}=\{{s}_{1},\,\mathrm{...,}\,{s}_{2N}\}$ corresponds to the 2*N* members of ${\bf{r}}=\{{r}_{1},\,\mathrm{...,}\,{r}_{2N}\}$ arranged in increasing order, and sgn(*x*) returns the sign of its argument, *x*. As we show in the Supplementary Material, Sec. 2, to obtain Eq. (13), one transforms the original many-fermion Hamiltonian to its representation in terms of bosonic creation and annihilation operators, places them in normal ordered form (where all creation operators lie to the left), and then truncates the unphysical excitations. Hence, by starting from a series of well-defined transformations, we have obtained a quantum mechanically exact representation of the fermionic matrix elements that is applicable to general many-fermion Hamiltonians.

## Index ordering: application to the Anderson and Hubbard models

The anticommutivity operator, $\hat{F}(j,k)$, clearly plays a vital role in our map (Eqs. (10) and (11)) since it allows one to transform fermionic operators to bosonic ones while avoiding the exponential scaling associated with the mapping of the many-body basis. However, since the scaling reduction comes at the price of introducing this nonlocal operator, it is important to consider how its influence manifests in mapped operators and systems. For example, consider the evaluation of an arbitrary quadratic operator, ${\hat{c}}_{j}^{\dagger }{\hat{c}}_{k}$, in its mapped form $\hat{F}(j,k){\hat{b}}_{j\beta }^{\dagger }{\hat{b}}_{j\alpha }{\hat{b}}_{k\alpha }^{\dagger }{\hat{b}}_{k\beta }$. In its mapped form, one can exploit the factorization of the many-body basis of bosons which allows for the calculation of the matrix elements of $\hat{F}(j,k){\hat{b}}_{j\beta }^{\dagger }{\hat{b}}_{j\alpha }{\hat{b}}_{k\alpha }^{\dagger }{\hat{b}}_{k\beta }$ with $\mathrm{2|}j-k|+2$ single-body inner products: 4 boson modes corresponding to the 2 indices *j* and *k* and the product of $\mathrm{2|}j-k|-2$ single-body bosonic operators in $\hat{F}(j,k)$. In contrast, if $\hat{F}(j,k)$ were absent, the matrix elements of the mapped operator would require only 4 single-body inner products. Hence, minimizing the presence of $\hat{F}(j,k)$ in mapped operators and Hamiltonians is advantageous in terms of efficiency. Below, we demonstrate how one can exploit the choice of index ordering in the JW transformation to curb the nonlocality of $\hat{F}$ and in some cases eliminate it completely. To illustrate this, we show how our map can be applied to the Anderson and Hubbard models, which are representative of fermionic systems belonging to the impurity and lattice families, respectively.

We start with the Anderson model,

15 $${\hat{H}}_{{\rm{And}}}=\sum _{\lambda }\,{\varepsilon }_{\lambda }{\hat{\tilde{n}}}_{\lambda }+\sum _{u,\lambda ,a}\,{\varepsilon }_{u,\lambda ,a}{\hat{n}}_{u,\lambda ,a}+U{\hat{\tilde{n}}}_{\uparrow }{\hat{\tilde{n}}}_{\downarrow }+\sum _{u,\lambda ,a}\,{t}_{u,\lambda ,a}\,[{\hat{c}}_{u,\lambda ,a}^{\dagger }{\hat{\tilde{c}}}_{\lambda }+{\hat{\tilde{c}}}_{\lambda }^{\dagger }{\hat{c}}_{u,\lambda ,a}],$$

where an impurity, which can accommodate an interacting pair of spin up and down electrons, is coupled to two leads per spin at (possibly) different temperatures and/or chemical potentials. Here, operators with an additional tilde $\{{\hat{\tilde{c}}}^{\dagger },\hat{\tilde{c}},\hat{\tilde{n}}\}$ correspond to the impurity, while all others correspond to the fermions in the leads, ${\hat{n}}_{u}={\hat{c}}_{u}^{\dagger }{\hat{c}}_{u}$ is the occupation number operator, *u* labels the single-particle orbitals that comprise the leads, $\lambda \in \{\uparrow ,\downarrow \}$ labels the spin, and $a\in \{{\rm{R}},{\rm{L}}\}$ distinguishes the right (*R*) from left (*L*) spin-dependent leads. The parameters of the Hamiltonian include the single-electron terms consisting of the impurity and lead state energies *ε*_*λ*_ and *ε*_*j*, *λ*_ and the coupling (impurity-lead hybridization) terms, *t*_*j*, *λ*_, connecting the impurity and lead states, while *U* is the two-body Coulomb term.

Using Eqs. (10) and (11), one can map the Anderson Hamiltonian, Eq. (15), as

16 $${\hat{H}}_{{\rm{And}}}\mapsto \sum _{\lambda }\,{\varepsilon }_{\lambda }{\hat{\tilde{\eta }}}_{\lambda }+\sum _{u,\lambda ,a}\,{\varepsilon }_{u,\lambda ,a}{\hat{\eta }}_{u,\lambda ,a}+U{\hat{\tilde{\eta }}}_{\uparrow }{\hat{\tilde{\eta }}}_{\downarrow }+\frac{1}{2}\sum _{u,\lambda ,a}\,{\hat{F}}_{\lambda ,a}\mathrm{(0,}u){t}_{u,\lambda ,a}[{\hat{\tilde{x}}}_{\lambda }{\hat{x}}_{u,\lambda ,a}+{\hat{\tilde{y}}}_{\lambda }{\hat{y}}_{u,\lambda ,a}],$$

where

17a $${\hat{\eta }}_{u}\equiv {\hat{m}}_{u,\beta }({\hat{m}}_{u,\alpha }+1),$$

17b $${\hat{x}}_{u}\equiv ({\hat{q}}_{u\beta }{\hat{q}}_{u\alpha }+{\hat{p}}_{u\beta }{\hat{p}}_{u\alpha }),$$

17c $${\hat{y}}_{u}\equiv ({\hat{q}}_{u\beta }{\hat{p}}_{u\alpha }-{\hat{q}}_{u\alpha }{\hat{p}}_{u\beta }),$$

and ${\hat{m}}_{j}$ is defined in Eq. (12). We emphasize that, since every fermionic creation and annihilation operator is mapped onto two coupled oscillators, quadratic operators, such as the occupation number operator, ${\hat{n}}_{u}\mapsto {\hat{\eta }}_{u}$, result in expressions that are quadratic in the oscillator occupation operators and quartic in the phase space variables, i.e., ${\hat{m}}_{u,\beta }({\hat{m}}_{u,\alpha }+1)=({\hat{q}}_{u,\beta }^{2}+{\hat{p}}_{u,\beta }^{2}-1)({\hat{q}}_{u,\alpha }^{2}+{\hat{p}}_{u,\alpha }^{2}+1)/4$. By using the Matsubara-Matsuda transformation to map the spins in Eq. (5), one can construct a phase space representation that yields only quadratic terms in the phase space variables. However, as we show in the Supplementary Material, Sec. 3.2, such a map would not be exact. We also note that only the final term in Eq. (16) contains the nonlocal operator $\hat{F}$, since, for occupation number operators, the mapped product of creation and annihilation operators on the same site trivially removes the nonlocal component, regardless of ordering.

**Figure 1:**
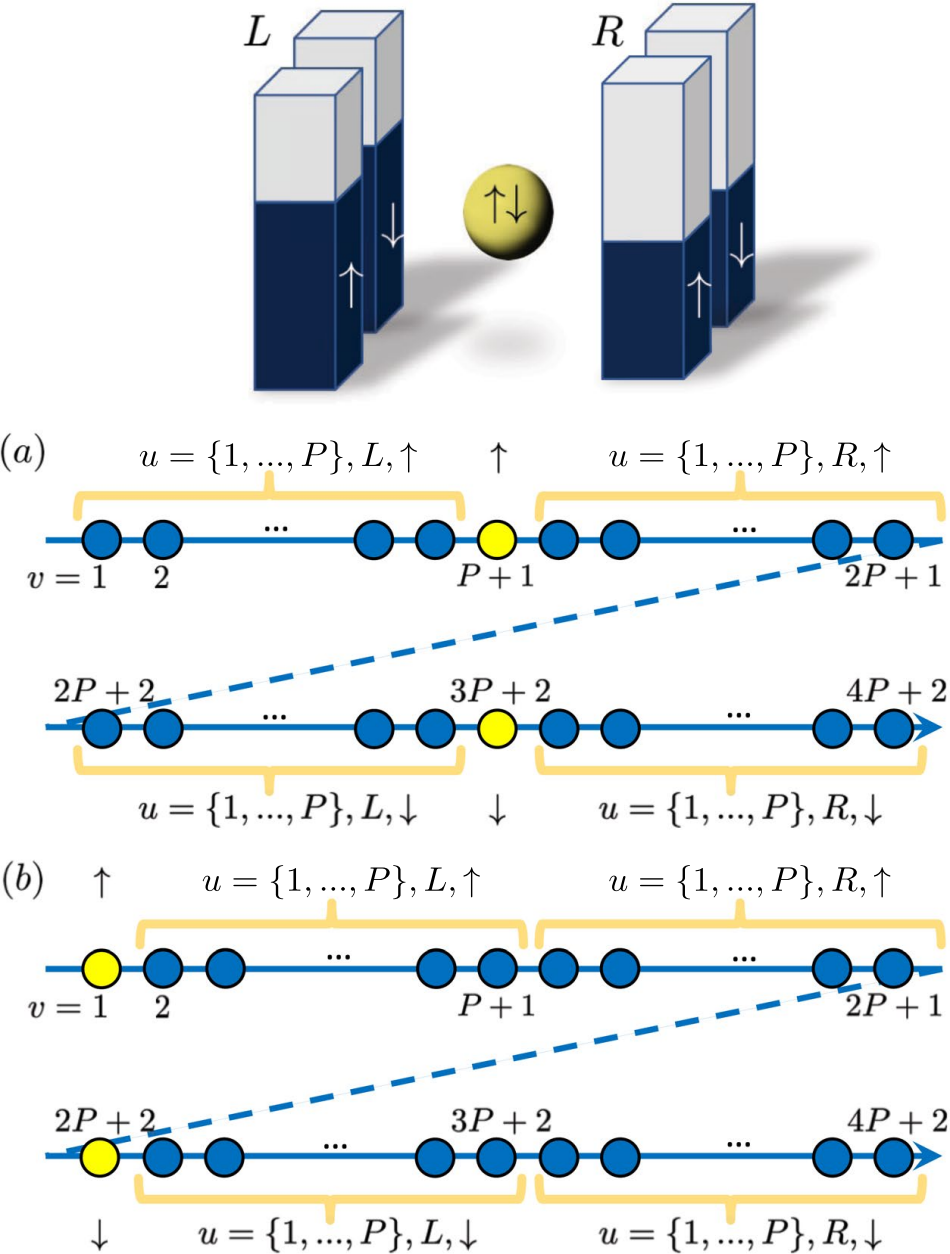
Index ordering options for the Anderson model.

To obtain the mapped version of any Hamiltonian, one must perform the JW transformation, which maps all the single-particle states that constitute the system to a 1D ordered spin chain i.e., with a single index. Hence, in the context of the Anderson model, it is necessary to choose an index ordering that both collapses the labels over the single-particle identifier, fermion spin, and lead label onto a single index, and minimizes the extent to which the nonlinear operator $\hat{F}$ appears in the mapped Hamiltonian. To achieve this, we arrange the single-particle orbitals along a chain corresponding first to *λ* = ↑, starting with the *a* = *R* lead states, continuing with the impurity state, and ending with the *a* = *L* lead states. A similar arrangement is then chosen for the *λ* = ↓ states. In principle, the leads correspond to infinitely large sources of electrons. However, in practice, one discretizes them into a suitably large number, *P*, of single-particle orbitals. This results in the 1D indexing: for the lead states $v=({\delta }_{a,R}-{\delta }_{a,L})u+P+1+\mathrm{(2}P+\mathrm{1)}{\delta }_{\lambda ,\downarrow }$ and for the impurity orbitals $v={\delta }_{\lambda ,\uparrow }(P+\mathrm{1)}+{\delta }_{\lambda ,\downarrow }\mathrm{(3}P+\mathrm{2)}$. This indexing is depicted in Fig. 1a.

Because there are no spin-flip terms (e.g. ${\hat{c}}_{u,\lambda ,a}^{\dagger }{\hat{c}}_{u^{\prime} ,\lambda ^{\prime} ,a^{\prime} }$) in this Hamiltonian, this indexing allows one to separate the spin chain into two parts which are not connected by the nonlocal operator $\hat{F}(v,\,v^{\prime} )$. This ability to separate the spin chain means that $\hat{F}(v,\,v^{\prime} )$ can be written as simply ${\hat{F}}_{\uparrow ,a}\mathrm{(0,}\,u)$ or ${\hat{F}}_{\downarrow ,a}\mathrm{(0,}\,u)$ (i.e., containing only connections within a particular lead) without introducing cross terms.

To illustrate the importance of the choice of index ordering, one can consider the effect of instead starting with *λ* = ↑, going from the impurity to the *a* = *R* and *a* = *L* orbitals, and then continuing with the *λ* = ↓ orbitals in the same fashion (Fig. 1b). While this choice of index ordering still provides a formally exact mapping, it results in the hopping terms connecting the impurity and right leads being modified by the occupations in the left leads, i.e., ${t}_{u,\lambda ,R}{\hat{F}}_{\lambda ,L}(0,\,P){\hat{F}}_{\lambda ,R}(0,\,u)$. Hence, it is important to consider which ordering leads to the simplest version of the mapped operators.

We now turn to the 1D Hubbard model,

18 $${\hat{H}}_{{\rm{Hub}}}=\sum _{u}\,{U}_{u}{\hat{n}}_{\uparrow ,u}{\hat{n}}_{\downarrow ,u}+\sum _{u,\lambda }\,{t}_{u,u+1}^{(\lambda )}[{\hat{c}}_{\lambda ,u}^{\dagger }{\hat{c}}_{\lambda ,u+1}+{\hat{c}}_{\lambda ,u+1}^{\dagger }{\hat{c}}_{\lambda ,u}],$$

which consists of a chain of sites that can accommodate interacting spin up and down fermions with nearest neighbor coupling. Here, $\lambda \in \{\uparrow ,\downarrow \}$ is the spin index, *u* is the spatial index of the sites along the Hubbard chain, ${t}_{u,u+1}^{(\lambda )}$ is the one-electron nearest neighbor hopping term, and *U*_*u*_ is the two-body Coulomb repulsion term, analogous to *h*_*j*, *k*_ and *U*_*jk*, *lm*_ in Eq. (1), respectively.

Using Eqs. (10) and (11), one can map the Hubbard Hamiltonian, Eq. (18), as

19 $${\hat{H}}_{{\rm{Hub}}}\mapsto \sum _{u}\,{U}_{u}{\hat{\eta }}_{\uparrow ,u}{\hat{\eta }}_{\downarrow ,u}+\frac{1}{2}\sum _{u,\lambda }\,{t}_{u,u+1}^{(\lambda )}[{\hat{x}}_{\lambda ,u}{\hat{x}}_{\lambda ,u+1}+{\hat{y}}_{\lambda ,u}{\hat{y}}_{\lambda ,u+1}],$$

where ${\hat{\eta }}_{\lambda ,u}$, ${\hat{x}}_{\lambda ,u}$ and ${\hat{y}}_{\lambda ,u}$ are defined in Eq. (17a–c). In practice, the infinite Hubbard chain is truncated to *P* sites.

**Figure 2:**
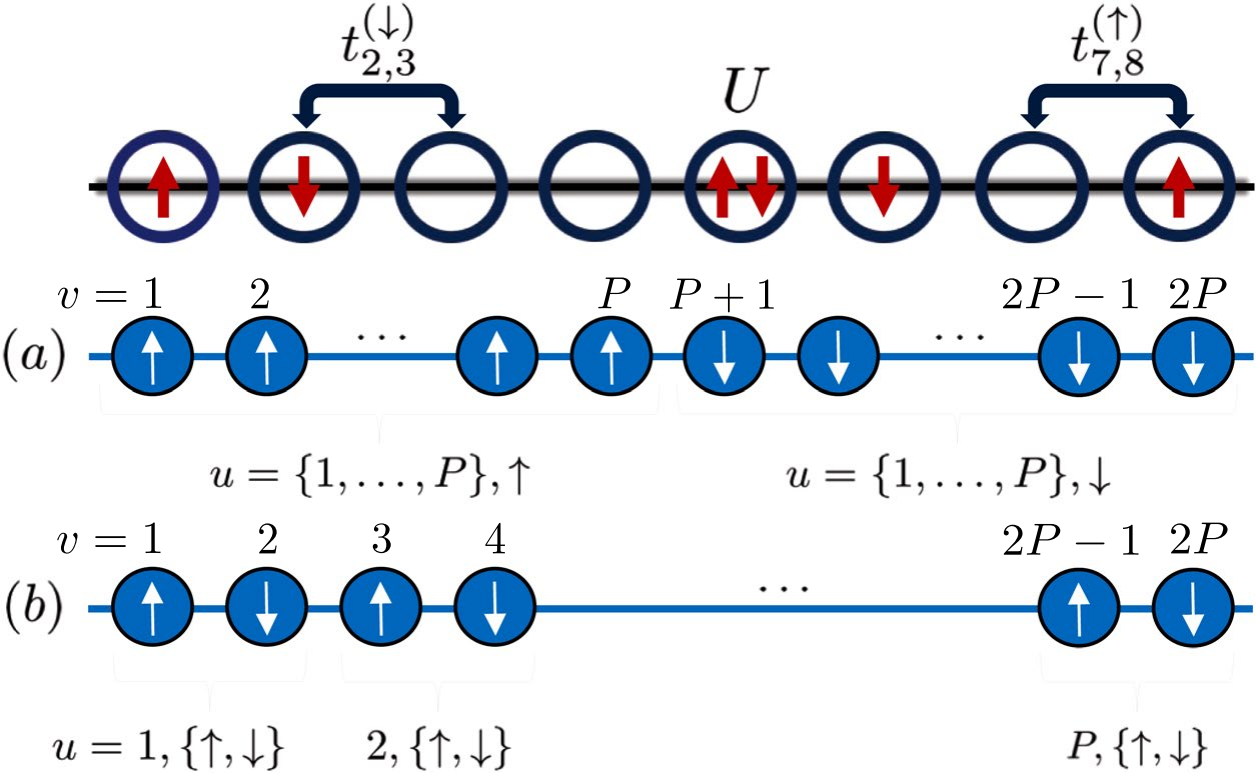
Index ordering options for the 1D Hubbard model.

As for the Anderson model, the first term, which contains only occupation number operators, does not have nonlocal contributions, $\hat{F}$, regardless of the index ordering, while the hopping term generally contains them. However, one can remove them with a judicious choice of index ordering. To do this, we choose the fermion indices as $v=u+{\delta }_{\lambda ,\uparrow }P$, where $u\in \mathrm{\{1,2,}\,\mathrm{...,}\,P\}$ labels the site number along the Hubbard chain. Following this indexing prescription, shown in Fig. 2a, one can exploit the nearest neighbor coupling in the 1D Hubbard model to obtain a mapped Hamiltonian that is completely free from the influence of the nonlocal operator $\hat{F}$. As for the Anderson model, we then transform from *v* to the original indices *u* and *λ*, leading to Eq. (19).

In contrast, if one were to order the chain according to the scheme shown in Fig. 2b, intercalating the up and down spins as one sweeps from left to right across the chain, i.e., $v=\mathrm{(2}u-\mathrm{1)}{\delta }_{\lambda ,\uparrow }+2u{\delta }_{\lambda ,\downarrow }$, one would not be able to eliminate all the nonlocal terms. Indeed, this choice would result in a renormalization of the hopping terms by an operator that tracks the occupation of one of the two neighboring sites with a fermion of the opposite spin, i.e., ${t}_{u,u+1}^{(\uparrow )}\mapsto {t}_{u,u+1}^{(\uparrow )}\,f({\hat{m}}_{\downarrow ,u,\beta }-{\hat{m}}_{\downarrow ,u,\alpha })$ and ${t}_{u,u+1}^{(\downarrow )}\mapsto {t}_{u,u+1}^{(\downarrow )}\,f({\hat{m}}_{\uparrow ,u+1,\beta }-{\hat{m}}_{\uparrow ,u+1,\alpha })$. It is thus noteworthy that when the transformation introduced here is employed with a judicious choice of indexing, one can rewrite the 1D Hubbard model entirely as a continuous bosonic Hamiltonian devoid of the nonlocal operator $\hat{F}$, which is consequently free from the complexities arising from the sign flips associated with fermionic anticommutivity.

## Conclusions

In conclusion, we have derived a map that expresses fermionic creation and annihilation operators in terms of continuous, Cartesian phase space variables. Importantly, this representation captures the exact matrix structure of the many-fermion problem. We have then shown how one can apply the map introduced here to the Anderson and 1D Hubbard models in a way that minimizes, and in some cases completely eliminates, the nonlocal fermionic anticommutivity operator, $\hat{F}$.

Finally, it is worth contrasting this work with previous approaches that can in principle exactly describe many-fermion problems in continuous phase space. These fall into two distinct categories. The first is based on bosonic coherent states and exploits the variational principle to describe the dynamics of a many-body antisymmetrized wavefunction ansatz^67–69^, at the price of poor scaling with system size^69^. The second is based on fermionic coherent states and focuses on second-quantized operators instead of the wavefunction^27–30,32,33^. However, when considering individual fermionic creation or annihilation operators, it becomes necessary to use Grassmann variables, which are objects of high computational complexity whose phase space distributions can present interpretational difficulties that require subsequent mapping to a complex number phase space^29,33,70^. In contrast, our approach is fully compatible with bosonic and spin coherent states^71,72^, which circumvents the difficulties posed by Grassmann variables while also benefiting from the improved scaling that arises from mapping the individual fermionic creation and annihilation operators rather than the many-body wavefunctions.

The *exact* Cartesian representation of fermionic creation and annihilation operators introduced here provides a starting point to employ methods that exploit classical-like trajectories to calculate static and dynamic quantum properties of many-fermion systems, including exact path integrals and approximate semiclassical and quantum-classical theories. In addition, our work provides a connection between bosonic and fermionic systems, which is necessary for treating fermionic problems using continuous-variable quantum computing techniques^73^. This mapping enables the systematic development of a hierarchy of trajectory-based quantum dynamical methods of varying cost and accuracy that can be tuned to the scale and requirements of the physical problem under consideration. By rendering the many-fermion problem in a form that is compatible with broadly applicable trajectory-based methods, our approach thus provides an avenue for the study of many problems that are currently inaccessible to existing methods, including those where fermionic degrees of freedom are coupled to complex and anharmonic nuclear motion and spins.

## Supplementary Material

See the Supplementary Material (SM) for a complete analysis of the mathematical procedures and transformations necessary for the derivation of Eqs. (10) and (11) and an exploration of the utility of other spin-to-boson transformations for the derivation of alternative fermion-to-Cartesian variable maps. In particular, in SM Sec. 1, we briefly review the necessary background of the Schwinger theory of angular momentum and demonstrate how it is exact at the matrix element level but not the operator level. In SM Sec. 2, we provide a derivation of how one can use the JW transformation and the Schwinger theory of angular momentum to obtain a fermion-to-boson map that exactly reproduces the matrix structure of the many-fermion problem. In this section, we demonstrate that all the spurious excitations that arise as a result of the Schwinger mapping are eliminated when the physical basis is used to evaluate the matrix elements, which ensures that *both* the forward and backward fermion-to-spin-to-boson transformations are well defined. In SM Sec. 3, we show that, for the purpose of obtaining an exact and practical Cartesian mapping of fermionic operators, two alternative spin-to-boson transformations that one may consider suffer from serious deficiencies.

## Electronic supplementary material

Supplementary Material
